# The Elusive *Trypanosoma cruzi* Disperse Gene Protein Family (DGF-1)

**DOI:** 10.3390/pathogens12020292

**Published:** 2023-02-10

**Authors:** José Luis Ramírez

**Affiliations:** Instituto de Estudios Avanzados, Caracas, Venezuela and Universidad Central de Venezuela, Caracas 1080, Venezuela; ramjoseluis@gmail.com

**Keywords:** chagas disease, surface proteins, disperse protein family, *Trypanosoma cruzi*

## Abstract

Chagas disease, caused by *Trypanosoma cruzi* infections, is included in the group of neglected diseases, and efforts to develop new therapeutic or immunoprevention approaches have not been successful. After the publication of the *T. cruzi* genome, the number of molecular and biochemical studies on this parasite has increased considerably, many of which are focused on families of variant surface proteins, especially trans-sialidases, mucins, and mucin-associated proteins. The disperse gene protein 1 family (DGF-1) is one of the most abundant families in the *T. cruzi* genome; however, the large gene size, high copy numbers, and low antibody titers detected in infected humans make it an unattractive study target. However, here we argue that given the ubiquitous presence in all *T. cruzi* species, and physicochemical characteristics, the DGF-1 gene family may play and important role in host-parasite interactions.

## 1. Introduction

*Trypanosoma cruzi*, the etiological agent of American Trypanosomiasis or Chagas disease, has proven to be a big challenge for those working on the molecular biology and genomics of the parasite. The repetitive nature of the *T. cruzi* genome, the presence of cell hybridization events, variations in ploidy, and chromosomal size polymorphisms have led to heated debates about the nature of its population dynamics [1]. Nonetheless, *T. cruzi* is a pathogenic entity, and depending on geographic locations and routes of infection, the symptoms in the human host vary [2]. Chagas is currently reported from the south of the United States to Argentina, and due to human migration, cases are also registered in non-endemic regions of Europe and Asia [3]. Attempts to develop new effective drugs or vaccines have failed, and the only drugs for treatment are nifurtimox and benznidazole. The parasite’s resilience and variability rely on its very dynamic genome, making it necessary to search for new ways to control this parasite [4]. The Disperse Gene Family (DGF-1) ranked fifth among the most repeated gene families of the *T. cruzi* CL Brener genome with 565 gene copies and 136 pseudogenes. Additionally, members of the family are among the largest genes of the parasite [5]. Despite having been discovered more than 30 years ago [6], the role of DGF-1 remains a mystery. Here, we summarize the most relevant findings concerning this gene family, provide some clues about its potential function, and discuss the incongruences between the DGF-1 transcriptome and proteome results [7].

## 2. The Discovery of the DGF-1 Family

Wincker et al. (1990) [6] discovered the DGF-1 family while working on the genome of the *Didelphis marsupialis T. cruzi* strain Dm28, reporting 220 members ranging in length from 10 to 12 Kbp and representing about 1% of the parasite’s genome. In Northern blot experiments with pulse-field resolved chromosomes, it was found that the DGF-1 genes were spread throughout the genome and did not have internally repeated sequences. Interestingly, these authors postulated that, given the dispersion of the family and the large gene size, they could participate in inter- and intrachromosomal recombination events, generating chromosomal size polymorphisms. In a second work [8], the same authors registered abundant DGF-1 transcripts in the replicative epimastigote forms of the parasite and sequenced a 10 Kbp gene that was dubbed DGF-1.1. Based on the presence of cysteine rich motifs spread along the gene, they hinted at a potential role of this protein as a receptor, and the presence of two tripeptides RGD suggested potential interactions with the host cell. In DNA hybridization experiments with other trypanosomatids such as *Phytomonas* sp., *Leptomonas samueli*, *Blastocrithidia* sp., *Crithidia fasciculata,* and *Trypanosoma rangeli*, these authors reported that the family was exclusive of *T. cruzi* strains. However, DGF-1 genes were later found in whole genome sequencing of *T. rangeli* strains Sc-58, Coachi, and M80 [9,10] and more recently in the African stercoraria trypanosomes *T. theileri* [11] and *T. grayi* [12].

## 3. General Molecular Characteristics of DGF-1 Proteins 

**Figure 1:**
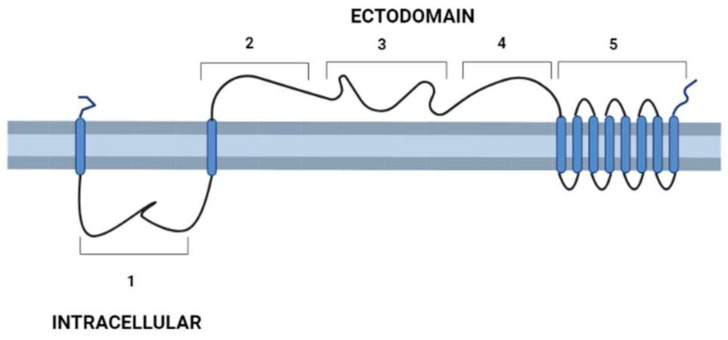
Schematic representation of *T. cruzi*’s DGF-1.2 protein in the cell membrane. Cell membrane cytoplasmic-extracellular topology predicted by UNIPRO. Numbers mark domains predicted by different structure-prediction software. 1. β-sheet structure (aa 60–140) identity to Human Galectin 7 2–3 (SWISSPRO); pectin-lyase superfamily IPRO11050 (aa 882–987) (InterPro); Regions 2, 3, and 4 with several structural predictions based on sequence identity, structure similarity, and amino acid folding: Complement component C9 receptor (aa 1189–1227) (aa 1584–1622) (SWISS-PRO); Notch ligand (Panther prediction) (aa 1192–1621); pectine lyase-fold (aa 1670–1898) (Alphafold-InterPro); arabinofuranosyltransferase aftd2 from mycobacteria (aa1993–2170) structure prediction confidence 96% (Phyre-2); 4. Threonine-rich region (aa 2800–2927) PROSITE, and structure of tailspike protein gp49 from pseudomonas phage2 lka1 88% confidence (Phyre-2), epitopes from reference [21] are located in this region; 5. Transmembrane helices region DGF1_14, DGF_1.5 (InterPro). Blue cylinders: Transmembrane domains. Graph made with BioRender.

After the description of the first DGF-1 gene (DGF-1.1) [6], other members of the family were characterized [13,14,15], whose most relevant features are: the presence of eight to nine transmembrane hydrophobic helices at their C-end that might serve as anchors to membranes six epidermal growth factor-1-like (EGF-1), and one EGF-2-like signature regularly spaced approximately 400 aa apart; and lectin binding motifs. There are also integrin-like sequences suggesting a cell surface location [13,14,15] and a possible role either interacting with other cells or as signal transduction receptors. These integrin-like motifs are closer to the primitive integrins (protointegrins) of *Saccharomyces cerevisiae*, *Entamoeba histolytica*, *Candida albicans,* and *Dyctiostelium*, having in common the RGD tripeptide mentioned above [6,16]. Approximately half of the family members have a canonical signal peptide, and all members lack a GPI anchor site [13]. A topology model of DGF-1.2 inserted in the cell membrane summarizing some features based on sequence homology searches, to which we added new information derived from recent advances in structural biology [17,18], is presented in Figure 1. The new structural and biological information revealed that the extracellular part of the protein has a sequence and structural identity with interactive domains such as pectin lyases, phage tail spikes, or receptors associated with virulence factors. The general topology also resembles those of well-studied receptors, with an external cytoplasmic interactive region and an internal cytoplasmic region which may be a signaling transmitting region [19,20]. 

## 4. DGF-1 Genealogy 

In their exhaustive study of the DGF-1 family in the CL Brener strain, Kawashita et al. (2009) [13], based on homology studies and the distribution of putative functional domains, divided the family into two main groups that included most gene members (66 and 51, respectively). The phylogenetic analysis also suggested that the family might have expanded through gene duplication events, but given the sequence similarity among members, these events were relatively recent or subjected to homogenization events. Also, the presence of parallel edges in the two main groups indicates that the family has experienced reticulate events such as recombination or gene conversion [13]. Two members of the family were clearly outgroups, namely XP 807429 (non-Esmeraldo-like haplotype type I group) and XP 816326 (Esmeraldo-like haplotype type II group), both lacking the pectin-lyase motif but having an immunoglobulin-like fold and a cysteine-proteinase inhibitor domain not present in the rest of the family. As mentioned before, members of the DGF-1 family have been found in *T. rangeli*, *T. theliri,* and *T. gayi*, and using genomic data, they were clustered in a distinct phylogenetic group [11]. 

**Figure 2:**
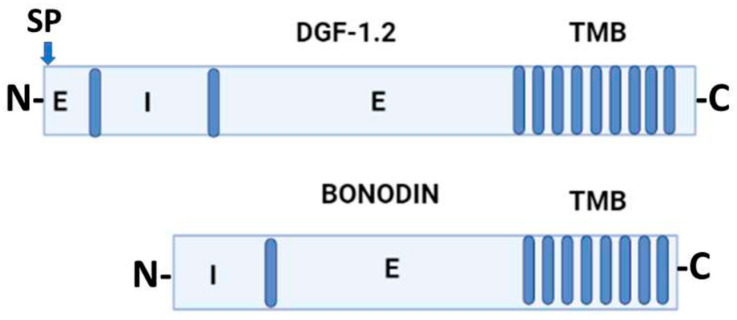
Schematic representation of *T. cruzi* DGF-1.2 protein and *Bodo saltans* Bonodin gene [17]. Barrels represent transmembrane regions; SP, signal peptide; N and C protein terminals; I, intracellular regions; E, extracellular regions. Graph made with BioRender.

Jackson et al. (2016) [22] proposed that DGF-1 genes appear to be innovations from more ancestral genes present in free-living organisms included in the genus *Bodo*, generically designated as Bodonin genes. Bodonin is defined as a multicopy gene family of transmembrane glycoproteins. The typical Bodonin gene has seven transmembrane anchors at the C-terminal, preceded by an extracellular domain, which in turn is preceded by an intracellular domain. The overall organization of the Bonodin and DGF-1.2 genes is shown in Figure 2. 

## 5. Chromosomal Distribution and DGF-1 Copy Numbers

DGF-1 gene organization was also revealed by Olivares et al. (2000) [23], who, while analyzing sequences of large *T. cruzi* BAC recombinants, reported DGF-1 members intermingling with the L1Tc retrotransposon. Later, Kim et al. (2005) [14] reported DGF-1 copies in the subtelomeric regions of BAC-telomere recombinants of the CLBrener strain. Interestingly, as in the case of Olivares et al. (2000) [23], the DGF-1 copies were surrounded by genes and pseudogenes of the trans-sialidases, retrotransposon hotspot sequence (RHS) families, and retrotransposon elements. Despite this seemingly unstable environment, the DGF-1 copies were uninterrupted ORFs. 

The subtelomeric location of DGF-1 has been confirmed in different *T. cruzi* strains [24,25,26], but there is a considerable variation in copy numbers among strains [24]. Berna et al. (2018) [26] found that DGF-1 genes were often clustered with other repeated families in what they called “disrupted chromosomal regions”. Following this idea, then, subtelomeres are part of these regions and are likely to be involved in *T. cruzi* chromosomal gene variation dynamics [14,25,27]. A very interesting observation made by de Bezerra de Araujo et al. [28] is the presence of chromosomal replication origins in subtelomeric DGF-1 members; this location could produce frontal collisions with the transcriptional machinery running in the direction of the telomere and cause chromosomal instability [29], chromosomal breaks, and recombination events leading to genetic variability.

## 6. Possible Roles for DGF-1 Genes

Our homology and structural searches indicate that DGF-1 genes interact with other proteins as receptors or bidirectional signal transductors; this function is likely to be regulated by cyclic AMP or nucleoside analogs [30,31] and non-cAMP kinases. This assumption is stressed by the results of Bao et al. (2008) [32] using two-hybrid experiments, where they found one cAMP site and two putative “non-typical” phosphorylation sites in DGF-1 peptides of the *T. cruzi* HO 3/15 strain. Moreover, Atwood et al. (2007) [33] in a glycoproteome study detected a glycopeptide that hybridized with many DGF-1 genes, and asserted that without this type of technology, these proteins would not have been detected. These features, together with the in-silico predictions, highlight the possibility that DGF-1 genes interact with other proteins and that these interactions are regulated. 

### In Silico Studies in DGF-1 

Azuaje et al. (2007) [34] tested the behavior of the trans-sialidases and DGF-1 gene families of *T. cruzi* CL Brener using mutagenesis pressure simulations. The task involved taking members of each family, subjecting them to repeated cycles of mutations, and then comparing the outcome with the repertoire of real sequences from the *T. cruzi* genome [5]. By these means, they observed that DGF-1 members, contrary to those of the trans-sialidase family, were able to generate variation at very low mutation rates or gene conversion events. In other words, DGF-1 genes displayed very robust behavior against more dramatic changes involving intrafamily exchanges, and highlighted the possibility that DGF-1’s pseudogenes may contribute to the generation of genetic variability or serve as buffers to avoid it. The contributions of pseudogenes in a dynamic exchange with the DGF-1 genes may also explain why Kawashita et al. (2009) [13] could not detect differences in codon usage frequencies between pseudogenes and non-pseudogenes. 

In a different study, this time exploring the robustness of genes of the trans-sialidase and DGF-1 families to undergo mutations causing changes at the codon level, Azuaje et al. (2007) [35] found no significant changes among members of the TS family except for the MVar1 and the DGF-1 family. The first was the most volatile member of the TS family, while the second stood out as a family with very low volatility. In other words, MVar1 had the highest capability to vary under the immunological pressure exerted by the host, whereas DGF-1 had the lowest. The results in volatility agreed with Shannon’s entropy studies performed by Kawashita et al. (2009) [13], where despite the presence of four blocks of high variability (high entropy), in general, DGF-1 sequences were very well conserved. Furthermore, when these high-variable blocks were tested for positive selection, a neutral evolution and purifying selection were found for some of these blocks. In summary, evidence contradicts the idea that the DGF-1 family is a relevant part of the parasite variable surface determinants, and that its role is more likely related to cell-to-cell interactions or signal transduction.

Gonzalez et al. (2009) [36] using machine learning techniques reported that *T. cruzi* adhesins such as GP82 and Tc8 [37] evolved differently than bacterial or fungi adhesins. In the case of *T. cruzi* adhesins, they bind to host cell receptors, triggering responses in both directions but avoiding a strong host immune response. In the case of DGF-1, when classifiers derived from *T. cruzi* adhesins were used, all members of the family were identified as adhesin-like proteins. In Kawashita et al. (2009) [13] as well as Gonzalez et al. (2009) [36], XP 807429 and XP 816326 were identified as outgroup sequences.

Pseudogenes deserve a separate discussion, since their presence in such amounts in the CL Brener *T. cruzi* genome cannot be ignored if we assume as good readings the total number of pseudogenes for the large families, they total approximately 2000 copies, and some of them are transcribed, polyadenylated, and even found in polysomes [38]. Experiments in mammals and *Drosophila* demonstrated that degenerated genes are transcribed [39,40] and that they can regulate parental genes through siRNAs. In addition, some processed pseudogenes seem to have evolved into primate microRNA genes [41]. *T. cruzi* lacks the activity of essential genes for the siRNA machinery [42,43] thus the possibility is open to participation as generators or suppressors of variability or as regulators of gene expression by unknown means.

## 7. Expression of DGF-1 Genes

Chandaa et al. (2007) [44] placed DGF-1 proteins among the highest expressed genes in Trypanosomatids and linked these genes to significant contributions to *T. cruzi* codon, amino acid, and genomic variations. However, there are significant discrepancies between transcriptome and proteome studies.

### 7.1. Translation

At the protein level, evidence gathered from several laboratories confirmed the presence of DGF-1 proteins in all *T. cruzi* developmental stages [13,15,45,46]. Nonetheless, in the first whole genome proteome study in *T. cruzi* CL Brener [47], no hits were found for these proteins. As mentioned before, a whole glycoproteome approach [29] reported a glycopeptide matching several members of the DGF-1 family. Using a different approach, Kawashita et al. (2009) [13] biotinylated *T. cruzi* trypomastigotes surface proteins and, after recovering the proteins bound or not to streptavidin columns, used a monoclonal antibody against a DGF-1 peptide to examine both fractions. The assay revealed that a 250 KDa band was present in both fractions, and the streptavidin-bound fraction had a more complex band pattern with additional lower MW bands. Interestingly, when these authors used the same antibody to probe the proteins secreted into the conditioned medium, they found similar band patterns, thus suggesting that DGF-1 proteins are shed into the medium and processed to generate lower molecular bands. In a trypomastigote-amastigote differentiation follow-up both axenic or infected HeLa and myoblast cells, we probed *T. cruzi* cells with an immunopurified polyclonal antibody prepared against a peptide from the DGF-1.2 protein [15]. In this experiment, we found that the DGF-1.2 protein was mainly expressed in amastigote forms and the highest expression occurred at 14 h in the cell free system, and at 18 h in the infected mammalian cells. During the trypomastigote-amastigote transition, the fluorescent particles containing DGF-1.2 progressed towards the inner side of the parasite cell membrane and were later released into the medium or inside the mammalian host cells. When we analyzed the cell-free differentiation medium using Southern blot experiments after 20 h of differentiation, we detected protein bands above 220 KDa as well as lower MW bands. When these bands were analyzed by MADIT-OF mass spectrometry, all of them presented matches with DGF-1 genes. These results indicated that DGF-1 gene expression is regulated during the differentiation from trypomastigotes to amastigotes and that the protein is processed into lower MW bands. Using a totally different approach consisting of cell-fractionation of *T. cruzi* trypomastigotes, followed by one-dimensional gel electrophoresis and LC-MS/MS, Ulrich et al. (2011) [45] identified different peptides that matched at least 39 members of the DGF-1 family, suggesting that these proteins were located in an organelle population different from acidocalcisomes, glycosomes, reservosomes, lipid droplets, or endocytic vesicles in the different stages of *T. cruzi* and provided definitive evidence that DGF-1 proteins are also expressed in epimastigote forms. Consistent with these results, Brossas et al. (2017) [46] studied the *T. cruzi* secretome during the trypomastigotes-amastigotes transition in Vero cells registered 18 DGF-1 proteins by Nano LC±MS/MS technology. The authors did not state whether these were free proteins or if they were included in vesicles. In our unpublished EM results with gold-labeled DGF-1.2, we detected these proteins included in vesicles smaller than 22 µ shed by all *T. cruzi* developmental forms.

In conclusion, DGF-1 proteins are expressed at all stages of *T. cruzi* development, but especially during trypomatigote-amastigote differentiation, and eventually released from amastigotes as small bodies inside the mammalian cell.

### 7.2. Transcription 

In most organisms, there is little agreement between transcriptome and proteome studies [48,49]. Some authors argue that the discrepancies are mostly due to technical problems that will eventually be resolved with more advanced proteomic techniques, but at least in developmental stages of *Leishmania,* the correlation was nearly 60% [50]. In Brossas et al. (2017) [46] proteomic analysis of the *T. cruzi* secretome of trypomastigote forms on CL Brener and VD strains, DGF-1 was second among the top proteins detected. Contrarily, Callejas-Hernández et al. (2019) [51] in their whole transcriptome analysis of trypomastigote forms of the *T. cruzi* Silvio X10 strain did not report any DGF-1 transcripts.

In most assays focused on the detection of RNAs by qPCR and proteins using specific antibodies, there is better agreement [13,15,45,46,52] than in whole proteome and transcriptome techniques. Most proteomic techniques will underestimate or miss proteins with modifications like lipidation, glycosylation, etc., and in silico prediction reveals that DGF-1 has many of these modification sites. Due to recent advances in DNA sequencing techniques and the affordability of this type of study, the number of *T. cruzi* transcriptomics reports have increased considerably [51] (not all are mentioned here). On the other hand, most transcriptome techniques rely on the ability to trap polyadenylated RNAs after cell lysis, including or excluding the nucleus. As Pastro et al. (2017) [53] demonstrated, there is a nuclear accumulation of some RNAs in *T. cruzi*, which at a given differentiation stage are transported to the cytoplasm for translation. Additionally, trypanosomatid genes are polycistronically transcribed and polyadenylated (including pseudogenes). However, not all RNAs are translated, a fact that is defined in some cases by the regulatory control sequences in their 3’UTRs [54,55] or by sequestration in mRNA-binding-protein bodies (RBP) [56]. Scmidin et al. (2015) [57] claimed a better agreement between transcription and translation in *T. cruzi* by isolating polysomal mRNAs. A clear example of this type of problem is revealed when we compare the GenSeq transcriptome results of Berna et al. (2017) [58] with the follow-up study of the DGF-1.2 protein by Lander et al. (2010) mentioned above. In the first case, the authors did not observe DGF-1 mRNA expression differences in all *T. cruzi* developmental stages, whereas in the second, a step-by-step approach revealed a clear time-regulated expression of DGF-1.2 during the trypomastigote-amastigote transition. Berna et al. (2017) [58] warned that their results should be taken cautiously given the possibility of missassembly and annotation problems in the *T. cruzi* Dm28c strain genome. A different problem in most transcriptome works is the lack of synchronicity of *T. cruzi* cells a simple observation of parasites in culture, or parasites recovered from Triatoma bugs confirm the extensive pleomorphism exhibited by *T. cruzi*’s cells. Without denying the utility of whole approaches, it appears that at this stage, they lack the precision to study the fine tuning of *T. cruzi* differentiation processes. 

## 8. Conclusions 

Despite the wealth of information provided by the new omics and advances in immunology, the role of a prominent and ubiquitous protein family like DGF-1 remains unknown.

The large number of copies and gene sizes of the DGF-1 family represent an energetic burden for any parasitic organism. In addition, DGF-1 genes appear to be very robust, with little variation among duplicated genes, suggesting that the function of most members of the family ought to be essential for the parasite. Is this stability related to the function of the protein per se, and/or does it also have to do with the location of replication origins inside these genes? 

It would be tempting to speculate that, given that *T. cruzi* groups with African stercorarian parasites, which also have DGF-1 families, these proteins may play a role in contaminative transmission. Nonetheless, *Trypanosome rangeli*, a typical salivarian parasite, has many putative DGF-1 gene copies, but so far, the DGF-1 proteins reported for this organism are rather small [9]. 

In vitro differentiation experiments have revealed that the DGF-1 protein is released inside the mammalian host cells and might interact, affecting their defenses [15]. Our attempts to obtain immunological protection in mice injected with DGF-1.2 have failed, and this in consistent with Kawashita et al.’s (2009) failure to block the parasite invasion [13]. However, both peptides used in these experiments (aa 779–1030 and 876–1117, respectively) faced the intracellular domain of the protein (Figure 1), and it is likely that by using peptides based on the protein ectodomain we might obtain a better immunological response. Most infectivity and immunological studies in *T. cruzi* focus on surface proteins like trans-sialidase, mucin, and mucin-associated protein families, to which the parasite developed a confounding strategy of simultaneously exposing multiple immunogenic determinants to avoid an effective response [59]. Thus, we ought to redirect our attention to targets not involved in this type of interaction. 

An example supporting this idea is the recent discovery that the *Trypanosoma brucei* invariant protein gp65 (ISG65) is a C3b complement receptor [60]. This receptor is an important part of the innate immune system, and its role is central to the activation of the alternative, classical, and lectin pathways of complement. Therefore, among other possibilities, this interaction could block the cascade of events that lead to complement parasite lysis, thus allowing the parasite to pass the first host immunological barrier.

Proteins associated with *T. cruzi*’s infectivity are mostly expressed in the infective forms of the parasite, i.e., metacyclic forms from the insect guts, intracellular amastigote replicative forms, and blood metacyclic forms, all of which seem to be resistant to complement-mediated lysis. In the acute phase of the disease, once amastigotes burst out of the host cells, they are exposed to the bloodstream, where they activate the complement system and bind to the C9 terminal part of the complement cascade, but at the same time, the C5b-9 complexes bind to the cell membranes but do not get internalized.

Iida et al. (1989) [61] and Ramírez-Toloza and Ferreira (2017) [62] have proposed that *T. cruzi* amastigote-complement resistance can be accounted for by the “presence of unidentified specific inhibitors, homologous to a host factor that will prevent the incorporation of C8 and C9 on amastigotes” (Iida et al., 1989) [61]. An alternative explanation by the same authors is that the presence of hydrophobic domains on the parasite surface molecules (or that they are released by the parasite) serves as non-specific “traps” of nascent C5b-7 complexes. Could the hydrophobic domains in DGF-1 be some of these traps? Is DGF-1 one of the many factors preventing the action of the host complement system, or is it a signal transduction receptor? Additionally, in a comprehensive computational screening of the *T. cruzi* proteome aimed to identify eight proteins for a multiple-epitope vaccine, DGF-1 epitopes were included in the final vaccine. For this selection, epitopes were chosen according to their predicted high antigenic and immunogenic MHC class I, MHC class II, and B cell properties [21]. 

## 9. Future Directions

The information described herein suggests that the DGF-1 family participates in basic signaling or the interaction between the parasite and the mammalian host in a manner unrelated to the evasion by typical surface protein variants of the trans-sialidases or mucin families. Although the large gene size and multiple copies of the DGF-1 family are important obstacles for molecular approaches, we believe it deserves further study.
